# Herbal medicine for irritable bowel syndrome: An overview of systematic reviews protocol

**DOI:** 10.1097/MD.0000000000026364

**Published:** 2021-06-18

**Authors:** Hyejin Jun, Seok-Jae Ko, Keumji Kim, Jinsung Kim, Hwan-Su Jung, Jae-Woo Park

**Affiliations:** a Department of Internal Korean Medicine, Kyung Hee University Hospital at Gangdong, Dongnam-ro 892, Gangdong gu; b Department of Clinical Korean Medicine, Graduate School of Kyung Hee University, Kyung-Hee Dae-ro 26, Dongdaemun-gu; c Department of Gastroenterology, College of Korean Medicine, Kyung Hee University; d Department of Pediatrics, College of Korean Medicine, Sang-Ji University, Sangjidae-gil 80, Wongju, Gangwon-do, Republic of Korea.

**Keywords:** herbal medicine, irritable bowel syndrome, overview of systematic reviews, protocol

## Abstract

**Background:**

Irritable bowel syndrome (IBS) is a common disorder characterized by the recurrence of abdominal pain and changes in bowel habits. Owing to the limitations of conventional treatments, patients with IBS are often dissatisfied with the effect of treatment and have a poor quality of life. Herbal medicines (HMs) are frequently used for the treatment of IBS. This protocol was designed through an overview of systematic reviews (SRs), to investigate the safety and efficacy of HMs for treating IBS.

**Methods:**

SRs published up to May 2021 will be searched from the following 6 electronic databases: Medline (via PubMed), EMBASE, Cochrane Database of Systematic Reviews, Allied and Complementary Medicine Database, Oriental Medicine Advanced Searching Integrated System, and China National Knowledge Infrastructure database. SRs and/or meta-analyses on the use of HMs for IBS will be included in this overview. The effects of a placebo, no treatment, usual care, or conventional treatment will be compared with those of HMs. Two investigators will independently extract the data and assess the methodological and evidence quality for each main finding. The total clinical effectiveness rate will be measured as the primary outcome.

**Results:**

This overview is expected to provide data on the use of HMs for the treatment of IBS based on qualitative and quantitative syntheses of the included SR data.

**Conclusion:**

This overview will evaluate and propose the efficacy and safety of HMs for the treatment of IBS.

**Registration number:**

DOI 10.17605/OSF.IO/NT6WZ (https://osf.io/nt6wz).

## 1 Introduction

Irritable bowel syndrome (IBS) is a symptom-based functional gastrointestinal disorder with a global prevalence of 11.2%.^[1]^ According to the Rome IV criteria, IBS is diagnosed on the basis of recurrent abdominal pain related to defecation or in association with a change in stool frequency or form. Symptoms must occur at least once per week in the previous 3 months, with a duration of at least 6 months.^[2]^ The pathophysiology of IBS remains unclear, but it may involve dysregulation of gut motility, visceral hypersensitivity, inflammation, post-infection, microbiomes, food sensitivity, genetics, and psychosocial dysfunction.^[3]^ The primary treatment involves lifestyle correction and symptom management. For example, a diet low in fermentable oligosaccharides, disaccharides, monosaccharides, and polyols and exercise is recommended. Depending on the symptoms (diarrhea-dominant, constipation-dominant, or mixed), the use of laxatives or loperamide, followed by bile acid sequestrants and 5-hydroxytryptamine 3 antagonists, can appropriately treat IBS.^[3]^

Many patients treated with these drugs do not experience significant improvements in IBS symptoms or the quality of life and are concerned about potential side effects. Therefore, many patients with IBS want to try complementary and alternative medicines.^[4]^ Herbal medicines (HMs) have long been used in Asian countries because of their safety, and the Cochrane library, in 2006, concluded that some HMs may improve the symptoms of IBS.^[5]^ HMs contain various ingredients, and these can act on multiple targets with potential synergistic effects; HMs that have been used to treat IBS-related symptoms for centuries with satisfactory effects will be the optimal choice.^[6]^

There have been several systematic reviews (SRs) on the effectiveness of HMs on IBS,^[7,8]^ but there is no overview that systematically synthesizes the SRs and evaluates their quality of evidence. Therefore, the purpose of this overview is to evaluate the evidence on the efficacy and safety of HMs for the treatment of IBS, obtained from SRs.

This protocol has been registered on the OSF registries (registration number: DOI 10.17605/OSF.IO/NT6WZ, URL: https://osf.io/nt6wz).

## 2 Methods

### 2.1 Inclusion criteria

#### 2.1.1 Types of studies

We will include SRs that estimate the effectiveness and safety of HMs for the treating IBS. The SRs should consist of randomized controlled trials (RCTs) irrespective of whether a meta-analysis was conducted or not. SRs of animal studies and SRs that analyzed the differences in the effectiveness among HMs will be excluded.

#### 2.1.2 Types of participants

Studies that included patients with IBS, regardless of age, sex, or race, diagnosed using the ROME criteria or other criteria stated by the authors, will be included.

#### 2.1.3 Types of interventions

Studies involving any type of oral HMs, either an original composition or a modified one with some herbs added or removed, regardless of dosage will be included. The control group will employ a placebo of a HM, usual care, conventional treatment such as western medication or no treatment.

#### 2.1.4 Types of outcome measures

The primary outcome measure will be the total clinical efficacy rate. Secondary outcomes will be the IBS symptom severity score, symptom score of IBS, short-form health survey score as a quality-of-life score, incidence of adverse events during the treatment, or recurrence rate after treatment.

### 2.2 Data sources

The following databases will be searched from inception dates to May 2021: 4 English databases (Medline via PubMed, EMBASE, Cochrane Database of Systematic Reviews, Allied and Complementary Medicine Database), 1 Korean database (Oriental Medicine Advanced Searching Integrated System), and 1 Chinese database (China National Knowledge Infrastructure database). The search strategy for Medline is presented in Table 1. Modified search strategies will be applied to other databases.

**Table 1:** Search strategy used in Medline.

#1. irritable bowel syndrome [mh]
#2. traditional Chinese medicine [tiab]
#3. herbal medicine [tiab]
#4. herb∗[tiab]
#5. systematic review [tiab]
#6. meta-analysis [tiab]
#7. #2 OR #3 OR #4
#8. #5 OR #6
#9. #1 AND #7 AND #8

If only the protocol of an SR is searched for, it is included in the overview reference list. If the scope of an overview is narrower than that of a relevant SR, we will include only subgroups of primary studies that meet the inclusion criteria of the overview.

### 2.3 Study selection and data extraction

#### 2.3.1 Selection of studies

**Figure 1:**
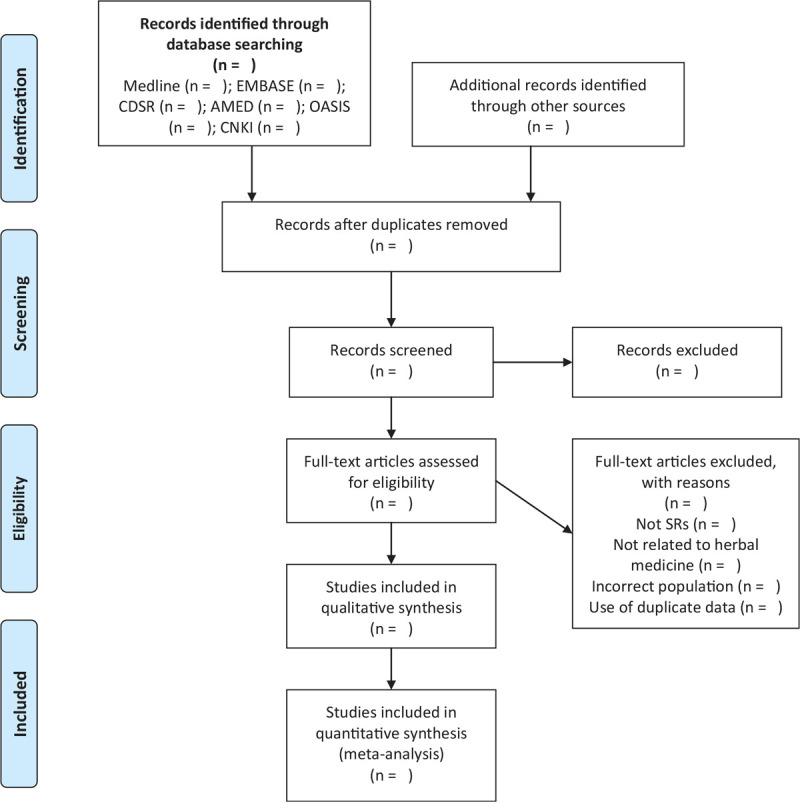
Flow chart depicting the literature screening and selection process. AMED = Allied and Complementary Medicine Database; CDSR = Cochrane Database of Systematic Reviews; CNKI = China National Knowledge Infrastructure Database; OASIS = Oriental Medicine Advanced Searching Integrat ed System; SR = systematic review.

Two reviewers (HJ and KK) will independently review the titles and abstracts of the studies that meet the inclusion criteria. All reviewers will receive education regarding the process and purpose of selection. The reasons for exclusion and number for excluded studies will be shown using a PRISMA flow chart (Fig. 1). Any disagreement will be resolved based on a discussion between the 2 reviewers. If necessary, the mediator (JWP) will intervene and resolve the disagreement.

#### 2.3.2 Data extraction and management

Two reviewers (HJ and KK) will independently extract the data and write the standard data extraction form, which includes basic study information such as the first author, publication year, written language, number of included studies and patients, IBS subtype, details of HM, control interventions, main results, and adverse events. Any disagreement will be resolved through discussion with a mediator (JWP).

### 2.4 Assessment of methodological quality

Two reviewers (HJ and KK) will assess the methodological quality of the included SRs using A MeaSurement Tool to Assess systematic Reviews (AMSTAR) 2.^[9]^ It is a validated tool that comprises 16 items, the responses to which can be “yes,” “partially yes,” or “no.” AMSTAR 2 does not generate an overall score but classifies the overall quality of each SR as “high,” “moderate,” “low,” or “critically low.” The 2 reviewers will discuss and resolve any arguments, and if necessary, a mediator (JWP) will intervene.

### 2.5 Data analysis

For the qualitative synthesis, data from each SR will be extracted in the form of odds ratio or risk ratio for dichotomous data and in the form of mean difference or standardized mean difference for continuous data with 95% confidence intervals.

For the quantitative synthesis of original RCTs, we will obtain the full text of the original RCTs included in the overview, exclude duplicate RCTs, and reanalyze the data using a meta-analysis approach. We will quantitatively synthesize the studies that use the same type of treatment, controls, and outcome measures. We will use the Review Manager program (version 5.3. Copenhagen: The Nordic Cochrane Centre, The Cochrane Collaboration, 2014) to perform statistical analyses. A random-effects model will be included in the meta-analysis. The heterogeneity of effect measures between the studies will be assessed using both the chi-square (*χ*^2^) test (*P* < .1 means statistical significance) and the *I*-squared statistic (*I*^2^ ≥ 50% means substantial heterogeneity).

If the data are available, we will assess a subgroup analysis according to type of herbal medicine and IBS subgroup (diarrhea-dominant, constipation-dominant, or mixed). Furthermore, if more than 10 studies are included in the meta-analysis, we will also conduct evidence of publication bias using funnel plots.

### 2.6 Assessment of quality of evidence

Two review authors (HJ and KK) will independently assess and report the quality of evidence for the main outcomes obtained from quantitative synthesis of original RCTs using the Grading of Recommendations, Assessment, Development, and Evaluation tool.^[10]^ It evaluates 5 main factors: risk of bias, inconsistency, indirectness, imprecision of results, and probability of publication bias. The quality of evidence will be graded on a 4-point scale: “very low,” “low,” “moderate,” or “high.”

### 2.7 Ethics and dissemination

This protocol is for overview of SR therefore ethical approval is not required. The results of this overview will be published in peer-reviewed journals or presented at relevant conferences.

## 3 Discussion

IBS is a disorder that affects a patient's quality of life, but conventional medical treatment does not fully exert sufficient therapeutic effect. Therefore, there have been steady attempts to treat IBS with HMs, and several SRs on the effectiveness of HM for treating IBS have already been published. However, there is no overview that systematically synthesizes the evidence presented in these SRs. Therefore, we will evaluate the evidence obtained from SRs on the efficacy and safety of HMs for treating IBS in this overview. In addition, we hope that our overview will support further research on IBS.

## Acknowledgments

This study was supported by the project “Development of Korean medicine clinical practice guidelines” of the Guideline Center for Korean Medicine, National Institute for Korean Medicine Development (project number: HF20C0051).

## Author contributions

**Conceptualization:** Hyejin Jun, Seok-Jae Ko.

**Data curation:** Hyejin Jun, Seok-Jae Ko, Keum-Ji Kim, Jin-sung Kim, Hwan-Su Jung, Jae-Woo Park.

**Formal analysis:** Jinsung Kim.

**Investigation:** Keum-Ji Kim, Jin-sung Kim, Hwan-Su Jung.

**Methodology:** Keum-Ji Kim, Jin-sung Kim, Hwan-Su Jung.

**Resources:** Jae-Woo Park.

**Writing – original draft:** Hyejin Jun.

**Writing – review & editing:** Seok-Jae Ko.
